# Supplementary material for: CC Genotype of GNAS c.393C>T (rs7121) Polymorphism Has a Protective Effect against Development of BK Viremia and BKV-Associated Nephropathy after Renal Transplant
Source: Pathogens. 2022 Oct 1;11(10):1138. doi: 10.3390/pathogens11101138 (PMC9609707; doi:10.3390/pathogens11101138)
Supplement: Supplementary file 1 [file pathogens-11-01138-s001.zip › pathogens-1905317-supplementary.pdf]

**Supplementary Table S1.** Results of univariate and multivariate analyses identifying risk factors and protective factors for development of *de novo* anti-HLA DSAs among 436 patients after renal allograft transplant.

| Variable | Patients with *de novo* anti-HLA DSAs n=51 | Patients without *de novo* anti-HLA DSAs n=385 | Univariate relative risk (95% CI) | *P* value | Multivariate relative risk (95% CI) | *P* value |
|---|---|---|---|---|---|---|
| Women, n (%) | 18 (35) | 165 (43) | 0.82 (0.54-1.17) | 0.30 | | |
| Previous transplants, n (%) | 12 (24) | 40 (10) | 2.27 (1.26-3.89) | **0.007** | 2.88 (1.42-5.83) | **0.003** |
| Preformed anti-HLA antibodies, n (%) | 22 (43) | 142 (37) | 1.26 (0.75-2.10) | 0.39 | | |
| Class I, n (%) | 8 (16) | 81 (21) | 0.75 (0.38-1.38) | 0.37 | | |
| Class II, n (%) | 4 (8) | 23 (6) | 1.31 (0.48-3.38) | 0.60 | | |
| Preformed anti-HLA DSAs, n (%) | 8 (16) | 30 (8) | 2.01 (0.97-3.97) | **0.06** | 1.19 (0.52-2.71) | 0.68 |
| MM (A/B), n (%) | 48 (94) | 314 (82) | 1.15 (1.03-1.24) | **0.03** | 3.85 (1.18-12.54) | **0.026** |
| MM (DR), n (%) | 40 (78) | 272 (71) | 1.11 (0.92-1.27) | 0.25 | | |
| ABO-incompatible transplant, n (%) | 3 (6) | 30 (8) | 0.75 (0.25-2.17) | 0.63 | | |
| Autoimmune disease as cause of ESRD, n (%) | 2 (4) | 16 (4) | 0.94 (0.24-3.46) | 0.94 | | |
| *GNAS* CC genotype, n (%) | 43 (84) | 276 (72) | 1.18 (1.00-1.32) | **0.056** | 1.94 (0.91-4.14) | 0.085 |
| Use of MMF/MPA, n (%) | 46 (90) | 316 (82) | 1.01 (0.96-1.19) | 0.15 | | |
| MMF level, mean (range) | 2.3 (0.1-22.0) | 2.5 (0.0-18.0) | | 0.44 | | |
| BK viremia, n (%) | 10 (20) | 91 (24) | 0.83 (0.45-1.42) | 0.52 | | |
| BKV-associated nephropathy, n (%) | 5 (10) | 25 (6) | 1.51 (0.61-3.54) | 0.38 | | |

BKV, BK virus; CI, confidence interval; DSA, donor-specific antibody; eGFR, estimated glomerular filtration rate; ESRD, end-stage renal disease; HLA, human leukocyte antigen; MM, mismatch; MMF, mycophenolate mofetil; MPA, mycophenolic acid.

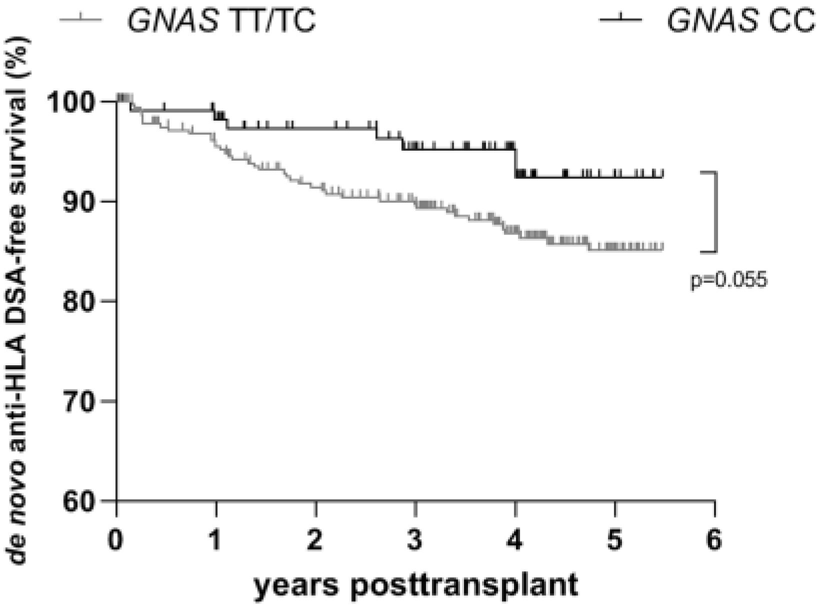


| | | | | | | | |
|---|---|---|---|---|---|---|---|
| *GNAS* TT/TC | 319 | 305 | 293 | 288 | 281 | 278 | 278 |
| *GNAS* CC | 117 | 115 | 114 | 112 | 111 | 110 | 110 |

**Supplementary Figure S1.** Survival of 436 renal allografts according to development of *de novo* anti-HLA DSAs and *GNAS* genotype during 5-year follow-up after transplant (*P*=0.055). BKV, BK virus; DSA, donor-specific antibody; HLA, human leukocyte antigen.
